# Antidepressant-Like Activity and Molecular Docking Analysis of a Sesquiterpene Lactone Isolated from the Root Bark of *Ximenia americana* (L.)

**DOI:** 10.1155/2024/6680821

**Published:** 2024-02-03

**Authors:** Tekeste Abebe, Ariaya Hymete, Mirutse Giday, Daniel Bisrat

**Affiliations:** ^1^Pharmacy School, College of Health Sciences and Medicine, Wolaita Sodo University, P.O. Box 138, Wolaita Sodo, Ethiopia; ^2^Department of Pharmaceutical Chemistry and Pharmacognosy, School of Pharmacy, College of Health Sciences, Addis Ababa University, P.O. Box 1176, Addis Ababa, Ethiopia; ^3^Aklilu Lemma Institute of Pathobiology, Addis Ababa University, P.O. Box 1176, Addis Ababa, Ethiopia

## Abstract

Depression, a global cause of disability and premature death, is often treated by traditional healers in Africa using medicinal herbs such as *Ximenia americana* (L.). With recent pharmacological studies showing the potential antidepressant properties of *X. americana* extract, this study aimed to evaluate the antidepressant-like effects of the compound(s) isolated from *X. americana* extract using the forced swim test (FST) and tail suspension test (TST) models predictive of depression. The extracts, administered orally within a dose range of 100–400 mg/kg, notably decreased the immobility time in both the FST and the TST. The most significant reduction occurred at the highest dose of 400 mg/kg, with a decrease of 117.66 s in FST and 53.5 s in TST. However, this reduction in immobility was not linked to changes in movements, as observed in an open-field test (OFT), suggesting that the effect of the extracts was not due to activation of locomotion. Subsequently, a sesquiterpene lactone, dehydrocostus lactone (**1**) was isolated through solubility-based fractionation and column chromatography of the active root bark extract of *X. americana*. Dehydrocostus lactone (400 mg/kg) demonstrated a 46.50 s reduction in immobility time in the FST, which was comparable to the positive control, imipramine (30 mg/kg). With a highly favorable docking score of −8.365 kcal/mol on an antidepressant target, monoamine oxidase A (MAO-A; pdb ID: 2BXS), dehydrocostus lactone (**1**) potentially outperforms the standard MAO-A inhibitor drug, isocarboxazid (−5.847 kcal/mol). Dehydrocostus lactone (**1**) displayed strong interactions involving hydrogen bond and hydrophobic and electrostatic interactions with specific MAO-A binding site residues. These findings highlight that the antidepressant-like activity of *X. americana* is partly attributed to the presence of dehydrocostus lactone. Additionally, it also supports the traditional medicinal use of the plant for treating depression.

## 1. Introduction

Depression, a pervasive psychological disorder, significantly impacts mood, physical well-being, and behavior, ranking as a leading cause of disability and premature death worldwide [1]. It affects around 10 to 20% of the global population [2] and manifests through negative emotions, reduced physical activity, feelings of helplessness, and even suicidal thoughts [3]. This condition correlates with low levels of essential monoamine neurotransmitters such as norepinephrine, serotonin, and dopamine in the brain and peripheral tissue [4, 5], attributed in part to the process of oxidative deamination catalyzed by the enzyme monoamine oxidase (MAO) in humans [6, 7].

Humans possess two types of MAO isoenzymes: monoamine oxidase A (MAO-A) and monoamine oxidase B (MAO-B) [8]. MAO-A predominately deaminates serotonin, norepinephrine, melatonin, and epinephrine, while MAO-B is responsible for deaminating phenethylamine and benzylamine. Dopamine, tyramine, and tryptamine are equally catalyzed by MAO-A and MAO-B [9]. Existing treatments for depression encompass diverse drugs, such as monoamine oxidase inhibitors (MAOIs), selective and nonselective monoamine reuptake inhibitors, selective serotonin reuptake inhibitors, and tricyclic antidepressants [10]. However, these medications often exhibit limitations, including adverse effects and partial effectiveness [11–13].

In Africa, herbal healers often use medicinal plants for the treatment of depression, including *Ximenia americana* [14–18], which is widely distributed in Africa [19]. While previous studies have highlighted the potential of *X. americana* extracts to alleviate depressive symptoms [20, 21], yet the compounds responsible for this effect remain unidentified. This led to the hypothesis that compound(s) in *X. americana* root bark extract could potentially inhibit MAO-A, thereby increasing the levels of crucial neurotransmitters such as noradrenaline, dopamine, and serotonin in both the brain and peripheral tissue. To address this hypothesis, the current study was undertaken to explore the antidepressant-like activity of the major compound(s) isolated from *X. americana* root bark extract using the forced swim test (FST) and tail suspension test (TST) models in mice. Molecular docking analysis was also performed on the isolated compound(s) to get some valuable insights into the interactions between the ligand-MOA_A_ at molecular level.

## 2. Materials and Methods

### 2.1. Chemicals and Instruments

The study utilized various chemicals and drugs, including imipramine (Torrent Pharmaceuticals, India); Tween-80 (BDH Chemicals Ltd, England); methanol (Carlo Erba reagents, France), chloroform and hexane (BDH, Poole, England); Analytical TLC (Silica gel 60 F254; 0.2 mm thick); and column chromatography (Silica gel F_60_ (70–240 mesh; Merck KGaA, Darmstadt, Germany). The instruments employed in this study were as follows: mass spectrum was generated using a UHPLC-MS system (Waters® Acquity, USA), and 1D- and 2D-NMR spectral data were recorded on a JNM-EX500 instrument (Jeol, Japan); other equipment used include a Rota Evaporator (Heidolph, Germany), Freeze Dryer (Hitachi, Japan), and a UV cabinet (CAMAG, UV Lamp 4).

### 2.2. Plant Materials

In March 2022, the root barks of *X. americana* were collected from the Kola area in Merhabete woreda, which is located 189 km to the north of Addis Ababa. The collected plant material was authenticated by *Mr*. *Melaku* Wondafrash (*botanist*) at Addis Ababa University's National Herbarium, College of Natural and Computational Sciences, and a voucher specimen (TA001) was deposited.

### 2.3. Extraction

The root barks of *X. americana* underwent a meticulous cleansing with distilled water to remove impurities, such as debris. Subsequently, they were dried in a shaded area with optimal ventilation and room temperature (25−27°C) for 15 days before being ground into a fine powder using a grinder. This resulting powder (600 g) was subjected to maceration in 80% methanol (1 L) for 72 h, intermittently shaken, and stirred. The marc (residue) was maceration three times, each time for 72 h, and then, the resulting filtrates were combined. The combined filtrates were concentrated by evaporating the solvent under reduced pressure using a Rota Evaporator. The resulting extract was carefully transferred into amber-colored vials and stored at 4°C until further use.

### 2.4. Isolation of Compound

The root bark extract of *X. americana* (62 g) underwent fractionation by solubility, yielding hexane (F1), EtOAc (F2), MeOH (F3), and water (F4) fractions. The hexane-soluble fraction (2 g) underwent further fractionation via a silica gel column. The column employed a gradient of EtOAc in hexane elution, starting with 100% hexane (F1-1 fraction; 50 mL), followed by ratios of hexane/EtOAc (9 : 1; F1-2), (8.5 : 1.5; F1-3), (1 : 1; F-14), and finally 100% EtOAc (F-1-5), each eluted with 50 mL. The fraction F1-2, eluted with hexane/EtOAc (9 : 1), showed a single spot on TLC under UV light of 254 nm using hexane/EtOAc (6 : 1) as the mobile phase. This fraction was designated as compound **1**, and its structure was determined through various spectroscopic techniques (^1^H, DEPT-135, ^13^C-NMR, HMBC, and ESI-MS).

**Dehydrocostus lactone** (**1**): white amorphous solid; *R*_*f*_ = 0.59 (TLC, hexane/EtOAc (6 : 1); ^1^H-NMR (*δ* ppm, 500 MHz): 1.28 (1H, *m*, H-4b), 1.87 (2H, *m*, H-7), 1.93 (1H, *m*, H-4a), 2.17 (1H, *m*, H-5b), 2.48 (1H, *m*, H-5a), 2.52 (2H, *m*, H-8), 2.88 (1H, *m*, H-9a), 2.98 (1H, *m*, H-6a), 2.99 (1H, *m*, H-3a), 3.98 (1H, *t*, 9.5 Hz, H-9b), 4.80 (1H, *brs*, H-12b), 4.92 (1H, *brs*, H-12a), 5.02 (1H, *brs*, H-11b), 5.19 (1H, *s*, 1 Hz, H-11a), 5.59 (1H, *d*, 3.5 Hz, H-10b), 6.13 (1H, *d*, 3.5 Hz, H-10a); ^13^C-NMR (*δ* ppm, 125 MHz): 31.2 (C-4), 32.2 (C-7), 33.6 (C-8), 37.8 (C-5), 46.1 (C-3a), 49.2 (C-6a), 53.2 (C-9a), 87.4 (C-9b), 109.3 (C-12), 112.7 (C-11), 120.8 (C-10), 141.6 (C-3), 151.2 (C-6), 153.5 (C-9), 172.4 (C-2); -ve mode ESI-MS: m/z = 229.3 [M-H]^−^; all NMR spectral data were consistent with data reported for the same compound [22].

### 2.5. Experimental Animals

Swiss albino mice of either sex, aged 6–9 weeks and weighing 20–30 g, were used in this study. In this study, female mice were also included because prior studies consistently demonstrated a higher occurrence of depression in women compared to men [23]. Additionally, many articles have also utilized female mice in their FST and TST tests [24, 25]. The mice were bred at the Animal House of the School of Pharmacy, Addis Ababa University, and provided with laboratory pellets and tap water. The animals were kept under standard light conditions (12/12 h light-dark cycle) and acclimatized for a week to the laboratory environment prior to the experiment. All experiments were carried out in the afternoon (4:00 PM), and the laboratory conditions, such as test room lighting, temperature, and noise level, were kept constant throughout the study [26]. An ethical approval letter was obtained from the Scientific and Ethics Committee of the School of Pharmacy, College of Health Sciences, Addis Ababa University (ERB/SOP/467/15/2023), following the guidelines of the Principles of Laboratory Animal Care [27]. The mice used in this study were maintained in the Pharmacology laboratory, School of Pharmacy, where the highest standards for the ethical treatment of animals were upheld. Throughout the study, the mice were not subjected to any unnecessary pain or distress. After the completion of the experiment, the mice were administered an anesthetic agent (pentobarbital sodium) to ensure their anesthesia before being euthanized through exsanguination. Once confirmed dead, the deceased mice were appropriately disposed of in the animal house's incinerator [28].

### 2.6. Grouping and Dosing of Animals

Five different groups of mice were randomly assigned with six mice in each group. Group I administered the vehicle (2% Tween-80 in distilled water) and served as the negative control, while Group II received the positive control (drug imipramine; 30 mg/kg). Group III-V received the extract dissolved in 2% Tween-80 at doses of 100 mg/kg, 200 mg/kg, and 400 mg/kg, respectively. The controls and test samples were orally administered 1 h prior to the experiment with the maximum volume being 10 mL/kg [29].

### 2.7. Forced Swim Test (FST)

The study assessed the antidepressant effects of *X. americana* root bark extract (100, 200, and 400 mg/kg), along with its isolated compound (100, 200, and 400 mg/kg), and imipramine (30 mg/kg) using the forced swim test (FST) protocol [24, 25, 30, 31]. In this test, mice underwent pretreated before being individually placed in a glass cylinder measuring 20 cm in height and 14 cm in diameter. The cylinder was filled with water up to a height of 10 cm and maintained at a temperature of 25°C. During a 6 min observation period, the total duration of immobility was noted. Immobility time was defined as the time interval during which the mice floated in water without any significant movement, except for necessary actions to keep their head or nostrils above the water surface. All mice involved in the experiment underwent a 15 min pretesting swim session 24 h prior to the actual FST to ensure their physical fitness. The efficacy of the treatments was compared against both the negative control and imipramine, which served as the positive control in this study.

(1) $$\begin{array}{c} \%\text{ reduction in time of immobility}=\frac{\text{Duration of immobility of negative control}-\text{Duration of immobility of test sample}}{\text{Duration of immobility of negative control}}. \end{array}$$

### 2.8. Tail Suspension Test (TST)

TST serves as a behavioral despair model for depression in mice, aiming to predict the potential antidepressant effects by reducing the duration of immobility caused by test substances [24, 25, 31, 32]. During this test, both the extract (100, 200, and 400 mg/kg) and the reference compound imipramine (30 mg/kg) were administered orally to the mice. Subsequently, the mice were suspended approximately 58 cm above the table's edge using adhesive tape placed roughly 1 cm away from the tip of their tails. Using a stopwatch, the total duration of immobility resulting from the tail suspension was recorded over a 6 min period. Mice were categorized as immobile only when they hung passively and were completely motionless [32, 33]. To ensure impartially, the observer present in the experimental room was unaware of the different treatments administered to the animals.

### 2.9. Open Field Test (OFT)

The OFT was utilized to assess the overall locomotor activity of the mice and to ensure that any observed effects of the extract (100, 200, and 400 mg/kg) and imipramine (30 mg/kg) were not false positives [31, 34, 35]. The OFT apparatus employed was a rectangular box measuring 68 cm × 68 cm × 45 cm. This box had a marked surface with horizontal and vertical lines, forming a grid of 16 squares. Additionally, a 60 W lamp was affixed above the box to provide illumination. To maintain cleanliness and eliminate any potential residue from prior tests, the inner surface of the box was meticulously cleaned using 99.8% alcohol and cotton, removing any fur, urine, and excrement. Following a 1 h treatment period, the mouse was placed in the center of the box. Its movements were then tracked and recorded via video for a duration of 6 min as it crossed both the central and peripheral squares within the grid.

### 2.10. Molecular Docking Experiment

This molecular docking study centered on exploring human monoamine oxidase A (MOA-A) as a potential target for antidepressants. Initially, dehydrocostus lactone (**1**) was assessed for its docking interactions with some potential targets. Additionally, as a point of reference, we examined isocarboxazid, currently used in treating severe depression and acting as a possible inhibitor of MAO-A [36].

#### 2.10.1. Protein Preparation

The X-ray crystallography structure of human monoamine oxidase A (MAO-A; PDB ID: 2BXS; resolution: 3.15 Å) complexed with clorgyline was downloaded from the Protein Data Bank (PDB; https://www.rcsb.org/structure/2BXS). The MAO-A structure consists of a 527 amino acid chain sequence, complexed with clorgyline (*N*-(3-(2,4-dichlorophenoxy)propyl)-*N*-methylprop-2-yn-1-amine) [37]. Using Maestro V13.5 (Schrödinger 2023-1) [38], the 3D structure of MAO-A protein in complex with clorgyline (PDB ID: 2BXS, chain A) was prepared using the protein preparation workflow module [39]. This involved correcting charges, bond orders, and atom types, removing water molecules beyond 5 Å from the het group, and filling in missing side chains and loops [40]. The OPLS4 force field was applied for energy optimization and steric hindrance removal [41].

#### 2.10.2. Ligand Preparation

ChemDraw Ultra (2019) was employed to draw the structure of compound **1** in MDL SDfile format. Ligand preparation was carried out using the Ligprep module of Maestro V13.5, Schrödinger Suite 2023-1. Different ionization states were generated at a pH of 7.0 ± 2.0 using Epik [42]. The stereoisomers of compound with specified chirality were generated by using OPLS4 forcefield. The prepared ligands were then docked to the prepared receptor by using the Glide docking tool, and the binding poses were analyzed based on the glide gscore.

#### 2.10.3. Receptor Grid Generation

We generated the receptor grid box to visualize the active site of the receptor for glide ligand docking. The grid was generated by selecting the cocrystal ligand to perform site-specific docking, with a 6.0 Å radius binding site. The van der Waals radii of the receptor atoms were set with a partial atomic charge scaling factor of 0.8 and a partial cutoff of 0.15 to soften the receptor's nonpolar parts.

#### 2.10.4. Ligand Docking

Using Glide software in extra precision (XP) mode, we docked compound **1** into the active binding site of the prepared protein. Prior to this, we docked the cocrystallized ligand into the protein's active site to predict binding affinity and molecular interaction. Docking scores in kcal/mol were used to analyze the binding pose. All the analyses and procedures were performed using the Schrodinger suite of programs.

### 2.11. Data Analysis

All data were analyzed using SPSS (Statistical Package for Social Sciences) software version 25. The results were presented as means with ±SEM, and ANOVA (one-way analysis of variance) was used to compare data between groups. The difference between the compared groups was considered statistically significant at a 95% confidence interval (*p* < 0.05).

## 3. Results

Powdered root barks of *X. americana* were subjected to maceration with 80% methanol, resulting in a reddish-brown amorphous solid (yield = 23.3%; w/w). Following this, the hexane-soluble portion of the root bark extract was further separated through silica gel column chromatography, leading to the isolation of a sesquiterpene lactone (**1**).

### 3.1. Structural Elucidation of the Isolated Compound (**1**)

Compound **1** was isolated as a white amorphous solid from the hexane fraction of the root bark extract of *X. americana*, with an *R*_*f*_ value of 0.59 on TLC using a solvent system of hexane/EtOAc (6 : 1). In the negative-mode ESI-mass spectrum (Figure S1), compound **1** gave a pseudomolecular ion at m/z = 229.3 [M-H]^−^, indicating a relative molecular weight (*M*_*r*_) of 230 mu. These data along with ^1^H and ^13^C-NMR data suggested that compound **1** had a molecular formula of C_15_H_18_O_2_.

**Figure 1:**
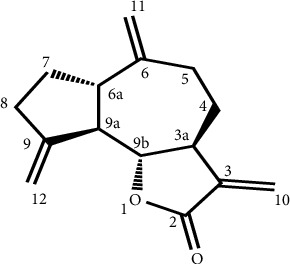
Structure of compound **1**.

Analysis of the ^1^H-NMR spectrum (Figure S2) of compound **1** showed the presence of three sets of exomethylene protons (*δ*4.80, 1H, *brs* and *δ*4.92, 1H, *brs*; *δ*5.02, 1H, *brs* and *δ*5.19, 1H, *d*, 1 Hz; *δ*5.59, 1H, *d*, 3.5 Hz; *δ*6.13, 1H, *d*, 3.5 Hz) as well as other important proton signals listed in the Materials and Methods section. The presence of fifteen carbon atoms was evident from the ^13^C-NMR spectrum (Figure S3) of compound **1**, consisting of four CH (*δ* 46.1, *δ* 49.2, *δ* 53.2, and *δ* 87.4), seven CH_2_ (*δ* 31.2, *δ* 32.2, *δ* 33.6, *δ* 37.8, *δ* 109.3, *δ* 112.7, and *δ* 120.8), three quaternary carbons (141.6, C-3; 151.2, C-6; 153.5, C-9), and one ester carbonyl (*δ* 172.4) from the DEPT-135 and DEPT-90 spectra. The remaining carbon signals are listed in the Materials and Methods section. Based on 1D-NMR (^1^H, ^13^C, DEPT-90, and DEPT-135 NMR), 2D-NMR (HMBC; Figure S4), and ESI-mass spectral data, compound **1** was unambiguously identified as 3,6,9-trimethylenedecahydroazuleno[4,5-b]furan-2(3H)-one, commonly known as dehydrocostus lactone (Figure 1). ^1^H and ^13^C-NMR chemical shifts of dehydrocostus lactone (**1**) were consistent with the one reported for the same compound [22].

### 3.2. Effect of the *Ximenia americana* Extract in Mice by the FST Model

**Figure 2:**
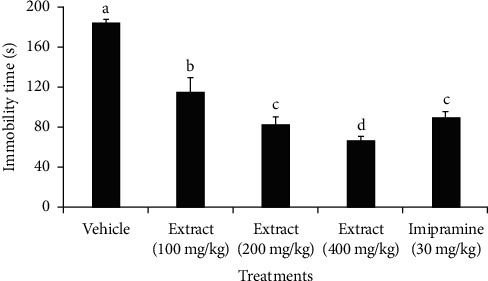
Effect of treatment with *Ximenia americana* root bark extract or standard drug (imipramine), given orally, on the immobility of mice in the FST. Values are presented as mean ± SEM; *n* = 6; vehicle: received 2% Tween-80 in distilled water; different letters indicate statistical significance by one-way ANOVA test followed by Tukey post hoc test (*p*<0.05).

In Figure 2, data from the FST model experiment illustrate the effects of varying doses of an extract on mice. Specifically, mice administered with doses of 100 mg/kg, 200 mg/kg, and 400 mg/kg extract displayed a significant decrease (*p* < 0.05) in the immobility duration compared to the negative control group. Notably, there was an observable trend: as the dosage increased, there was a corresponding decrease in immobility duration. Of the test doses, mice administered with the highest dose of the extract (400 mg/kg) exhibited a significantly greater reduction in immobility (*p* < 0.05) by 117.66 s (64.0%) compared to the negative control group. There were no apparent significant difference (*p* > 0.05) in immobility reductions between the 200 mg/kg extract (immobility reduction = 101.66 s) and the standard imipramine at 30 mg/kg (immobility reduction = 95.33 s).

Consequently, the extract demonstrated a dose-dependent response, highlighting varying reductions in immobility time at different doses. Specifically, the percentage reduction in immobility time for doses of 100, 200, and 400 mg/kg was 37.6%, 55.3%, and 64.0%, respectively. This dose-dependent trend indicates an increasing efficacy of the extract in reducing immobility, suggesting a potential correlation between dosage strength and the extract's impact on diminishing immobility in the tested mice.

### 3.3. Effect of *Ximenia americana* Extract in Mice by the TST Model

**Figure 3:**
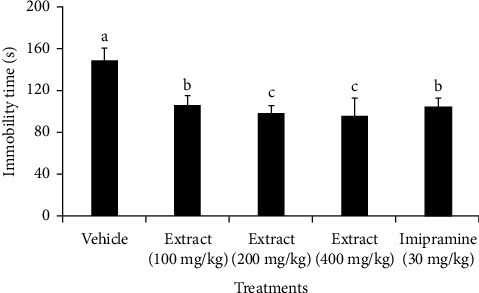
Effect of treatment with *Ximenia americana* root bark extract or standard drug (imipramine), given orally, on the immobility of mice in the TST. Values are presented as mean ± SEM; *n* = 6; vehicle: received 2% Tween-80 in distilled water; different letters indicate statistical significance by one-way ANOVA test followed by Tukey post hoc test (*p*<0.05).

Figure 3 presents the outcomes of the TST model experiment. The findings showed that mice administered with varying extract doses (100 mg/kg, 200 mg/kg, and 400 mg/kg) experienced a significant decrease (*p*<0.05) in immobility duration compared to the negative control group. Interestingly, while there was no significant difference (*p*>0.05) between the 200 mg/kg (immobility reduction by 50.84 s) and 400 mg/kg (immobility reduction by 53.5 s) extracts in reducing immobility, both doses produced a significantly higher reduction (*p*<0.05) than the 100 mg/kg dose and the standard drug, imipramine (30 mg/kg). Moreover, there were no substantial differences (*p*>0.05) in the reduction of immobility duration between the standard (imipramine 30 mg/kg) and the extract at 100 mg/kg. The percentage reductions in immobility time for the extract at doses of 100, 200, and 400 mg/kg were 29.3%, 34.2%, and 36.0%, respectively, revealing a dose-dependent effect. This suggests that the antidepressant-like effect of the extract tends to increase with higher doses, with a clear trend of increasing the activity as the dosage rises.

### 3.4. Effect of the *Ximenia americana* Extract on Locomotion in the OFT Model

Data obtained from the OFT are presented in Table 1. The results showed that both the standard and test samples did not exhibit a significant difference (*p* < 0.05) in the number of crossings when compared to the negative control. Additionally, there was no noticeable difference observed between different doses of the extracts and the standard. This suggests that increased motor activity did not contribute to the antidepressant-like action of the extract in either the FST or the TST. It confirms that the antidepressant-like effect is specific and not linked to increased motor activity.

### 3.5. Effect of Dehydrocostus Lactone on the Duration of Immobility in Mice in the FST Model

**Figure 4:**
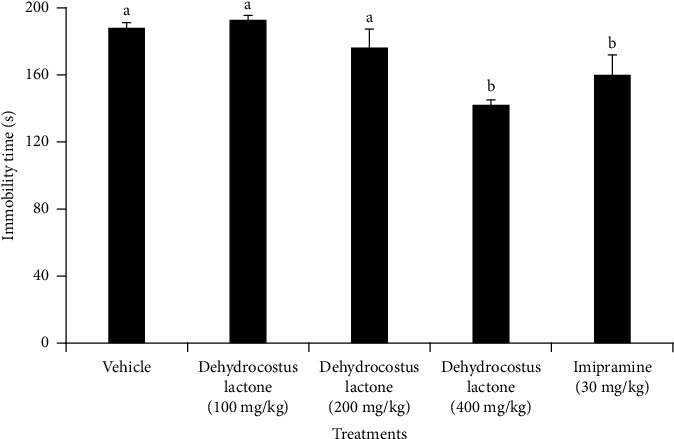
Effect of treatment with dehydrocostus lactone (**1**) or standard drug (imipramine), given orally, on the immobility of mice in the FST. Values are presented as mean ± SEM; *n* = 6; vehicle: received 2% Tween-80 in distilled water; different letters indicate statistical significance by one-way ANOVA test followed by Tukey post hoc test (*p*<0.05).

Antidepressant-like activity of the mice group treated with dehydrocostus lactone (**1**) is illustrated in Figure 4. Dehydrocostus lactone (**1**) was ineffective at doses of 100 mg/kg (−2.29%) and 200 mg/kg (6.44%) to show any marked decrease in immobility time compared to the negative control group. As the dose increased to 400 mg/kg, dehydrocostus lactone (**1**) showed a significant reduction (*p* < 0.05) in immobility time (24.62%, reduction) compared to the negative control group and the groups treated with 100 mg/kg and 200 mg/kg doses of the extract. Notably, there were no significant differences (*p* > 0.05) observed in immobility time between the groups treated with dehydrocostus lactone (**1**) (400 mg/kg) and the positive control, imipramine at 30 mg/kg.

### 3.6. Molecular Docking

Computer-aided drug design (CADD) approaches offer significant potential for understanding intricate interactions between ligands and receptors (enzymes) in biological systems [43, 44]. In this study, we employed a molecular docking method to investigate the interaction between dehydrocostus lactone (**1**) and MAO-A at the molecular level.

**Figure 5:**
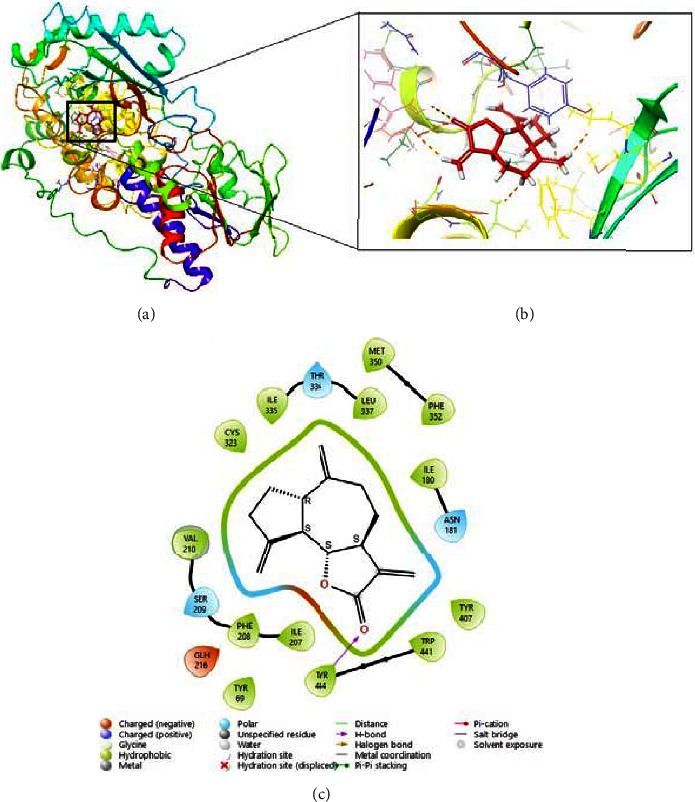
(a) 3D representation of dehydrocostus lactone (**1**) docked within the active site of MAO-A; (b) the 3D zoomed view of the dehydrocostus lactone (**1**) interaction (c) 2D model of dehydrocostus lactone (**1**) showing interactions with residues at the MAO-A enzyme.

Dehydrocostus lactone (**1**) was specifically docked into the active site of the MAO-A enzyme (PDB ID: 2BXS) using the Maestro V.13.5 software (Schrodinger 2023-1 Suite).  Figure 5 illustrates 2D and 3D representations of dehydrocostus lactone (**1**) docked within the active site of MAO-A. In the molecular docking study, dehydrocostus lactone exhibited a favorable docking score of −8.365 kcal/mol. This suggests a strong binding affinity, likely due to its capability to form a strong hydrogen bond with the amino acid residue Tyr444 located in the active site of the MAO-A enzyme (Figure 5). In addition, it has also hydrophobic and electrostatic interactions with specific residues in the binding site of the MAO-A enzyme, namely, Asn181, Cys323, Glh216, Ile180, Ile207, Ile335, Leu337, Met350, Phe208, Phe352, Ser209, Thr336, Tyr69, Tyr407, and Val210. Dehydrocostus lactone (**1**) displayed a high docking score of −8.365 kcal/mol on MAO-A, surpassing the standard MAO-A inhibitor drug, isocarboxazid (−5.847 kcal/mol; Figure S5). We can conclude that dehydrocostus lactone (**1**) potentially inhibits MAO-A from removing neurotransmitters from peripheral tissues. This highlights the potential promise of dehydrocostus lactone for further exploration through *in vivo* studies.

## 4. Discussion

Several depression treatments exist in the market, yet their success rates are around 50–60%, leaving a significant number of patients unresponsive to initial medication [45, 46]. Furthermore, most depression drugs cause intolerable side effects such as hypotension, arrhythmias, insomnia, and sexual dysfunction [47, 48]. This underscores that there is an urgent need for better, more tolerable, and more effective antidepressants. In this study, *X. americana* extract was given to mice orally tested for antidepressant-like effects using the FST and TST, standard animal tests for antidepressants. These tests involve subjecting mice to stressful situations, like being suspended in air or confined to swim in a restricted area. In these tests, increased immobility reflects a state of despair or lowered mood, which is indicative of depression in animals [49]. The results, outlined in Figures 2–4, indicate that the extract notably decreased the immobility time of mice in both the FST and TST, suggesting the antidepressant-like activity of the extract. The possibility that extract reduced immobility time in mice through a stimulant action was ruled out by using the OFT, as it measures the locomotor activity of animals [50]. Results from this experiment showed that the extract did not alter the locomotion of animals, further supporting the specificity of the effects observed in the FST and TST models [51].

Furthermore, the ability of the extract to lower immobility time in the FST was linked specifically to the presence of dehydrocostus lactone (**1**), a sesquiterpene lactone, as it significantly decreased immobility time compared to the vehicle-treated group (Figure 4). Dehydrocostus lactone (**1**) is identified here for the first time in *X. americana*, although it has been previously identified in other genera, such as *Echinops kebericho* [52] and *Parthenium hysterophorus* [53]. Sesquiterpene lactones have attracted significant attention from medicinal chemists due to their potential as antidepressants [54, 55]. Studies have indicated that the *α*, *β*-unsaturated lactone has the ability to increase the levels of monoamine neurotransmitters in the brain, which is a key feature linked with antidepressant effects [55, 56]. These activities are believed to be mediated by the lactone ring and *α*,*β*-methylene-*γ*-lactone in dehydrocostus lactone [57]. It is also noteworthy to mention that dehydrocostus lactone (**1**) exhibits various biological activities, including antibacterial and antifungal properties [52], as well as anti-inflammatory effects [58]. Additionally, terpenoids, to which dehydrocostus lactone belongs, have also been reported to play a significant role in the treatment of some behavioral disorders [59, 60]. In a recent study, Rashid et al. [61] discovered that the extract from *Melilotus officinalis* can alleviate pain in mice by influencing opioidergic, nitrergic, and muscarinic systems. This effect may have implications for mood regulation and depression due to its modulation of key brain neurotransmitters such as serotonin and norepinephrine. Bahrami et al. [62] also found that troxerutin, derived from the natural bioflavonoid rutin, when taken by pregnant female mice, increased superoxide dismutase (SOD), glutathione peroxidase (GPx), and total antioxidant status (TAS) levels in offspring and improved motor skills in mouse pups.

The monoamine theory is one of the commonly accepted explanations for depression. It suggests that depression can occur due to a decline in monoamine neurotransmitters such as norepinephrine (NE), serotonin (5-HT), and dopamine (DA), which are essential in regulating mood, anxiety, and depression [63]. In our study, we employed molecular docking methods, a crucial approach enabling thorough analysis at the molecular level. This technique provides theoretical insights beneficial for both *in vitro* and *in vivo* studies [64]. As illustrated in Figure 5, dehydrocostus lactone exhibited a strong docking score of −8.365 kcal/mol, indicating its capacity to potentially trigger antidepressant-like effects through the inhibition of monoamine oxidase (MAO-A). This inhibition occurs through strong interactions—hydrogen bonding, hydrophobic and electrostatic interactions—with specific residues in the binding site of the MAO-A enzyme. This led to our conclusion that dehydrocostus lactone (**1**) potentially acts as a monoamine oxidase A inhibitor (MAO-AI), leading to increased availability of neurotransmitters such as serotonin, norepinephrine, and dopamine in peripheral tissue [8, 9, 65] and reduces symptoms of anxiety and depression. With its notably high docking score of −8.365 kcal/mol on MAO-A, dehydrocostus lactone (**1**) potentially outperforms the standard MAO-A inhibitor drug, isocarboxazid (−5.847 kcal/mol). The significantly higher affinity of dehydrocostus lactone for MAO-A suggests it could offer enhanced therapeutic benefits, making it a good candidate for further research and potential development in treating depression.

Given its extensive presence throughout Africa, notably in Ethiopia, *X. americana* emerges as a readily accessible resource [19]. This wide distribution emphasizes the significant promise held by the extract derived from *X. americana* and its constituents for future potential herbal-based drug development.

## 5. Conclusions

In summary, this study marks the first identification of dehydrocostus lactone in *X. americana*. Notably, when administered at higher doses, dehydrocostus lactone displayed a significant reduction in immobility. In addition, through molecular docking analysis, it was observed that dehydrocostus lactone has the potential to inhibit MAO-A, although further experimental validation is necessary. These findings imply that the antidepressant-like property of *X. americana* could be partially attributed to the presence of dehydrocostus lactone in the root bark, aligning with the traditional medicinal use of this plant for treating depression. The widespread distribution of *X. americana* in Ethiopia underscores the substantial potential held by its extract and dehydrocostus lactone for future herbal-based drug development. Furthermore, the prospect of dehydrocostus lactone serving as a scaffold for synthesizing more potent compounds highlights its significance in drug development.

## Figures and Tables

**Table 1:** Results of the open field test in mice treated with *Ximenia americana* root bark extract.

Test samples, p.o	Number of crossings		
	Peripheral squares	Central squares	Total squares
Vehicle	69.17 ± 3.36^a^	7.17 ± 1.92^a^	76.34 ± 5.28^a^
Extract (100 mg/kg)	73.50 ± 2.14^a^	5.83 ± 1.13^a^	79.33 ± 3.17^a^
Extract (200 mg/kg)	75.50 ± 2.32^a^	8.00 ± 1.06^a^	83.50 ± 3.38^a^
Extract (400 mg/kg)	77.83 ± 2.62^a^	5.50 ± 1.17^a^	83.33 ± 3.79^a^
Imipramine (30 mg/kg)	72.17 ± 2.44^a^	8.50 ± 2.04^a^	80.67 ± 4.48^a^

*Note.* Values are presented as mean ± SEM; *n* = 6; vehicle: received 2% Tween-80 in distilled water; data were analyzed by one-way ANOVA followed by Tukey post hoc test; data followed by same letters within column are not significantly different at *p* > 0.05; p.o: per oral.

## Data Availability

The data used to support the findings of the study are available from the corresponding author upon request.

## Abbreviations

CC: Column chromatography
FST: Forced swim test
MAO-A: Monoamine oxidase A
MAOIs: Monoamine oxidase inhibitors
NSAIDS: Nonsteroidal anti-inflammatory drugs
OFT: Open field test
TLC: Thin-layer chromatography
TST: Tail suspension test.

## Supplementary Materials

Supplementary Materials Figure S1: (-Ve mode)-ESI-mass spectrum of dehydrocostus lactone (**1**); Figure S2: ^1^H-NMR spectrum of dehydrocostus lactone (**1**); Figure S3: (A) ^13^C-NMR, (B) DEPT-135; (C) DEPT-90 spectra of dehydrocostus lactone (**1**); Figure S4: HMBC spectrum of dehydrocostus lactone (**1**); and Figure S5: (A) 3D representation of isocarboxazid docked within the active site of MAO-A; (B) the 3D zoomed view of the isocarboxazid interaction; (C) 2D model of isocarboxazid showing interactions with residues at the MAO-A enzyme.
