# Supplementary figures and images for: Antidepressant-Like Activity and Molecular Docking Analysis of a Sesquiterpene Lactone Isolated from the Root Bark of Ximenia americana (L.)
Source: Evid Based Complement Alternat Med. 2024 Feb 3;2024:6680821. doi: 10.1155/2024/6680821 (PMC11390229; doi:10.1155/2024/6680821)

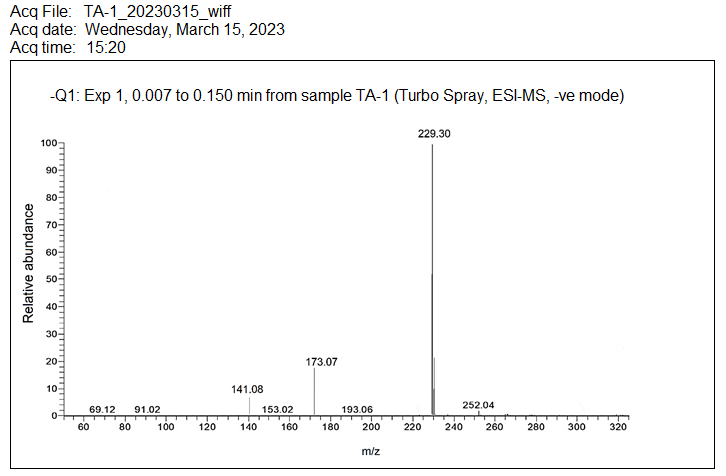


Figure S1


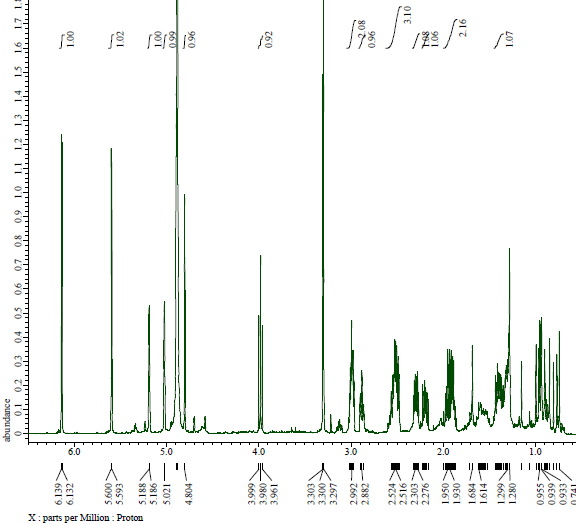


Figure S2


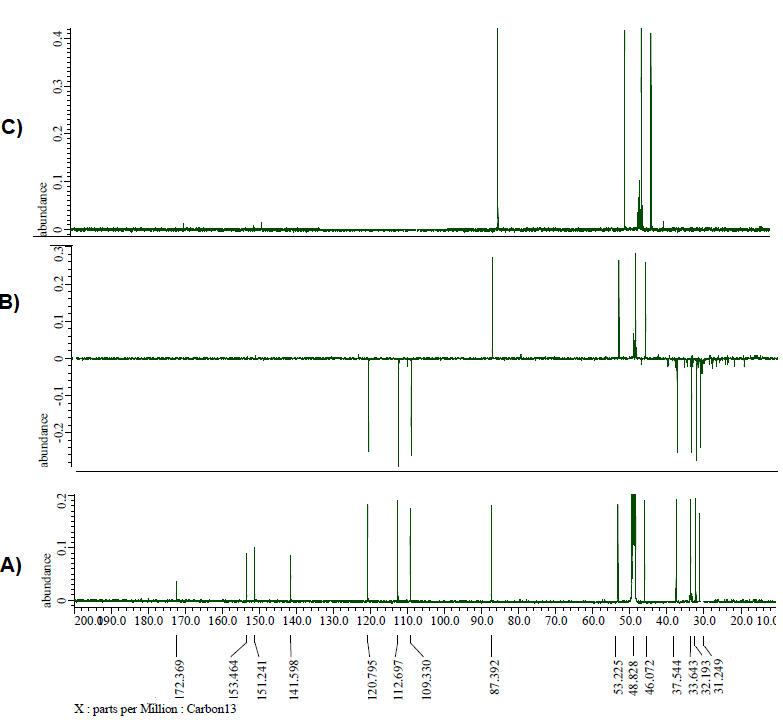


Figure S3

Figure S4


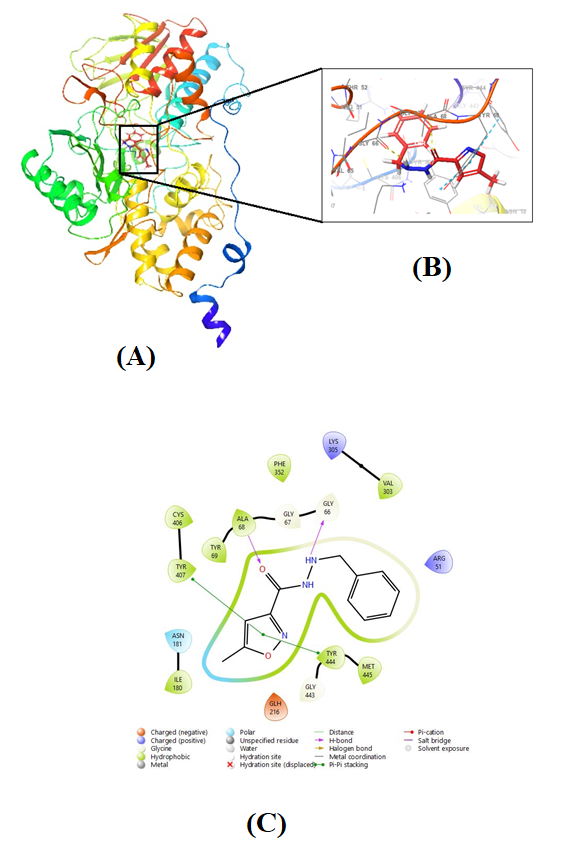


Figure S5

Supplement: Supplementary Materials — Figure S1: (-Ve mode)-ESI-mass spectrum of dehydrocostus lactone (1); Figure S2: 1H-NMR spectrum of dehydrocostus lactone (1); Figure S3: (A) 13C-NMR, (B) DEPT-135; (C) DEPT-90 spectra of dehydrocostus lactone (1); Figure S4: HMBC spectrum of dehydrocostus lactone (1); and Figure S5: (A) 3D representation of isocarboxazid docked within the active site of MAO-A; (B) the 3D zoomed view of the isocarboxazid interaction; (C) 2D model of isocarboxazid showing interactions with residues at the MAO-A enzyme. [file 6680821.f1.docx]
